# The Association Between Prenatal Maternal Selenium Concentration and Neurodevelopment in Early Childhood, Results from a Mother–Child Cohort Study

**DOI:** 10.1016/j.tjnut.2025.04.005

**Published:** 2025-04-11

**Authors:** Suman Ranjitkar, Ingrid Kvestad, Ram K Chandyo, Tor A Strand, Kjersti S Bakken, Manjeswori Ulak, Sandra Huber, Maria Averina, Merina Shrestha, Mari Hysing

**Affiliations:** 1 Department of Psychosocial Science, Faculty of Psychology, University of Bergen, Bergen, Norway; 2 Department of Pediatrics, Child Health Research Project, Maharajgunj, Kathmandu, Nepal; 3 Department of Research, Innlandet Hospital Trust, Lillehammer, Norway; 4 Community Medicine Department, Kathmandu Medical College, Sinamangal, Kathmandu, Nepal; 5 Center for International Health, University of Bergen, Bergen, Norway; 6 Women’s Clinic, Innlandet Hospital Trust, Lillehammer, Norway; 7 Department of Laboratory Medicine, University Hospital of North Norway, Tromsø, Norway; 8 Department of Clinical Medicine, The Arctic University of Norway, Tromsø, Norway

**Keywords:** maternal selenium, neurodevelopment, Bayley, early childhood, Nepal

## Abstract

**Background:**

Selenium (Se) is a micronutrient essential for human health and the developing brain. A few studies have demonstrated associations between maternal Se concentration and child neurodevelopment.

**Objectives:**

We aimed to describe Se status in pregnant Nepalese females and explore the association between maternal Se plasma concentration in early pregnancy and child neurodevelopment measured during early childhood in Nepalese children.

**Methods:**

The cohort study included 800 mother–infant dyads from Bhaktapur, Nepal. Blood samples from pregnant females were drawn within 15 wk of gestation and Se concentration was analyzed by inductively coupled plasma mass spectrometry. Child neurodevelopment was assessed at 6, 12, and 24 mo with the Bayley Scales of Infant and Toddler Development, 3rd edition (Bayley-3). We used linear mixed models to examine the association between maternal plasma Se concentration and Bayley-3 scores, adjusted for maternal age and socioeconomic status.

**Results:**

The mean (standard deviation) maternal plasma Se concentration was 74.8 μg/L (10.4 μg/L), and 290 (36.3%) pregnant females had Se concentration indicating deficiency (<71.1 μg/L). We found no significant association between maternal Se concentration and the Bayley-3 total *z*-score [Coeff. 0.002 (95% confidence interval: –0.007, 0.011)], and no associations between Se concentration and any of the Bayley-3 composite and subscale scores.

**Conclusions:**

Despite a substantial proportion of pregnant females with Se deficiency, maternal Se concentration was not associated with early childhood neurodevelopment in our study cohort of healthy pregnant Nepalese females.

## Introduction

Poor nutritional status during pregnancy can influence fetal nourishment [1] and impair growth and neurodevelopment during infancy [2]. Selenium (Se) is a micronutrient essential for several biological processes, particularly brain development and neurological function [3]. The richest sources of Se in foods are Brazil nuts, seafood, meat, poultry, and organ meats. Additional sources include cereals and dairy products [4]. There is a variation in Se concentration in soil according to geographical regions [5]. Se content in crops or plants is associated with the Se levels in the soil [6].

During pregnancy, the daily demand of Se for the fetus is ∼4 μg, whereas the basic recommended intake for pregnant females is 60 μg [7]. Due to its essential role in the fetal developing brain, Se requirements during pregnancy are increased [8]. Deficiency of Se during pregnancy can increase risk of gestational diabetes and hypertension, birth complications such as miscarriage and preterm birth, and low-birth weight (LBW) [9,10]. Although most of the low- and middle-income countries (LMICs) do not have proper documentation on Se status [11], some LMICs, such as India, Sri Lanka, and Nepal are shown to be Se-deficient areas [12,13]. However, in Nepal, the status of Se among females of reproductive age is not well known.

The brain contains 2.4% of the total Se in the body [14]. Selenoprotein-P (SELENOP) in the blood is responsible for Se transport around the body playing a vital role in the brain, especially within the olfactory bulb, cerebral cortex, hippocampus, and cerebellar cortex [3,15] representing potential mechanisms for Se to influence child development. In a study of 6-mo-old Norwegian infants, those with low maternal Se concentration (<71.1 μg/L) measured at 18 wk of gestation had lower neurodevelopmental scores measured by the screening tool Ages and Stages Questionnaire-3rd edition (ASQ-3) than those with higher Se concentrations [16]. Similarly, a Japanese study found positive associations between maternal concentrations of Se during mid/late-term pregnancy and children's ASQ scores [17]. Using the Bayley Scales of Infant and Toddler Development, 3rd Edition (Bayley-3), widely considered the reference standard for neurodevelopment assessments in infants and toddlers, studies have shown positive associations between maternal Se and neurodevelopment during the first 2 y of life [18,19].

Most of these studies are from Europe [16,18,19], a few from Asia [17,20], and other LMICs [21,22]. Furthermore, most studies have investigated Se deficiency during pregnancy and its impact on neurodevelopment in early childhood [[17], [18], [19],^23^]. One exception is a study conducted in Bangladesh [21]. In this 10-y follow-up, maternal Se concentration was positively associated with scores on a general ability test in children. However, child Se concentration was measured in hair and urine; there was an inverse association with cognitive abilities for those with Se concentrations >95th percentile. This indicates that elevated Se concentrations may have harmful effects on cognitive development. Moreover, in a study of Spanish children, an inverted U-shape relationship was observed between maternal Se concentration and cognitive abilities as measured by McCarthy Scales of Children's Abilities, suggesting that both low and high Se concentrations may be harmful to child neurodevelopment [24].

There is some evidence of sex differences in the association between maternal Se concentration and neurodevelopment. In the study from Bangladesh, a positive association between maternal Se concentration and psychomotor development at 18 mo was observed in females, but not in males [22]. Similarly, a positive association between cord-blood Se concentration and scores on the Wechsler Intelligence Scale for Children was found in girls, but not in boys [20]. On the basis of findings from a study in the United States, vitamin B_12_ could be another potential moderator between Se concentration and neurodevelopment [23]. Among mothers with B_12_ levels above the median, increased Se concentration by 1 IQR increased the odds of having a child diagnosed with autism spectrum disorder by 52%. A social gradient in Se status is prevalent [25,26] and maternal age can influence the Se status [27]. Neurodevelopment of children is associated both with maternal age [28] and socioeconomic status [29,30].

In LMICs, improving child neurodevelopment in future generations is a critical issue [31]. In Nepal, recent estimates show that 35% of the children are at risk of not reaching their developmental potential [32]. Still, few studies have been conducted on the relationship between maternal Se and neurodevelopment in LMICs.

The main aims of this study are to describe Se status among pregnant females in Nepal and to explore possible associations between maternal Se concentration measured in early pregnancy and neurodevelopment of their child aged at 6, 12, and 24 mo. We also aimed to assess whether these associations are moderated by the sex of the child and maternal vitamin B_12_ supplementation.

## Methods

### Study design and setting

**FIGURE 1:**
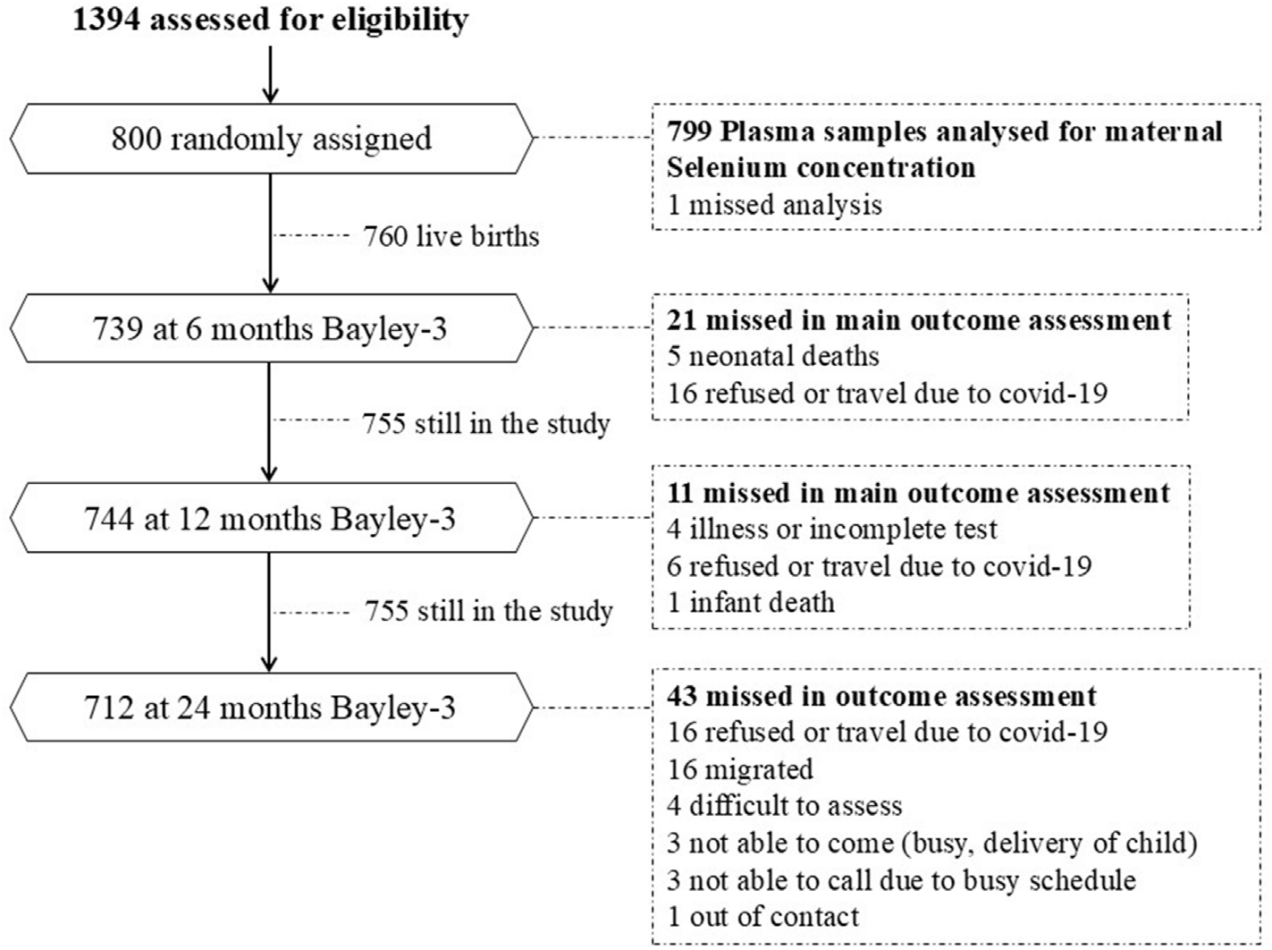
Study flow diagram for major activities from enrollment to 24 mo in Nepalese mothers and children.

The mothers and children included in the current study were originally participants in a double-blind randomized controlled trial entitled “Supplementation of vitamin B_12_ in pregnancy and postpartum on growth and neurodevelopment in early childhood: A Randomized, Placebo Controlled Trial” [33]. We randomly assigned 800 pregnant females (1:1 ratio) to daily supplementation of 50 μg oral vitamin B_12_ or a placebo from early pregnancy to 6 mo postpartum. During the first trimester, we also provided daily 400 μg of folic acid, and from the second trimester to 45 d postpartum, 500–1000 mg calcium and 60 mg iron as per national guidelines to all pregnant females. There was no measurable effect of vitamin B_12_ supplementation on growth and neurodevelopment in the children measured aged at 6 and 12 mo [34]. In this study, we included all participating females with a measurement of maternal Se concentration within 15 wk of gestation and their offspring (*n* = 767). Participant flow, including the reasons for lost follow-up and missing data, are presented in the flow diagram (Figure 1).

The study was conducted in Bhaktapur municipality in Bhaktapur district and its surrounding communities, located 15 km east of the capital city of Nepal. At the time of the study, the district had a population of 432,132 in 2021 with an annual population growth rate of 3.32%. Most of the people in this area are engaged in agriculture, small-scale business, service, or daily wage jobs.

### Procedure

Females from the general population were recruited early in pregnancy mostly through antenatal clinic-based surveillance between March 2017 and October 2020. Antenatal checkups (ANCs) are almost universal in the study area and all enrolled females could follow their ANC visits as per the recommendation of treating gynecologists.

We included pregnant females aged 20–40 y, no later than 15 wk of gestation calculated based on the last menstruation period with further confirmation by ultrasonography. The families should also plan to reside in Bhaktapur municipality and its surrounding areas during the study period. Females taking or planning to take multivitamins containing vitamin B_12_, who had chronic illnesses, severe anemia (Hb <7 g/dL), and/or a BMI lower than 18.5 kg/m^2^ and higher than 30.0 kg/m^2^, were excluded from the study.

All participants were followed every week until 6 mo postpartum for documentation of the compliance of supplementation, food intake, morbidities, and hospital visits. The participants were requested to visit the field site when the child aged 6, 12, and 24 mo for neurodevelopmental assessments. For the 24-mo visit, we asked for additional consent because the original trial was designed only for ≤12 mo of child age.

### Demographic and clinical information

Demographic and clinical information, including family socioeconomic factors, parental characteristics, and clinical and biological factors, were collected at enrollment. The socioeconomic status was presented by a socioeconomic index, the Water and sanitation, Asset ownership, Maternal education, and household Income (WAMI), based on variables reported by parents at the enrollment [35]. The WAMI is a composite measure that includes socioeconomic indicators related to safe drinking water and sanitation, the presence of household assets, maternal education, and household income. Details of the calculations are provided in Supplemental Table 1.

Caste/ethnicity was recorded as Brahmin, Chhetri, Newar, Gurung/Rai/Magar, Tamang/Lama, Chaudhari/Madhesi/Muslim, Dalits, and others. For this study, we categorized these into Newar, Brahmin/Chhetri, Indigenous groups (Gurung/Rai/Magar/Tamang/Lama), and others. Parent educational levels were defined as years of schooling. For the analysis, we dichotomized educational level based on completion of secondary school and above. The occupation was recorded as a housewife, agriculture, carpet worker, daily wage earner, business, government employee, services in the private sector, and foreign employment. For the present analyses, we dichotomized the variable into whether or not the mother had formal work. Those involved in agriculture, services, business, daily wage-based jobs and foreign employment were regarded as formal work, and housewives as no formal work. Birth weight was based on hospital reports and birth weight <2500 g was defined as LBW. The gestational age was also based on hospital reports and <37 wk was regarded as preterm birth.

### Laboratory procedure

At enrollment, ∼3–4 mL of venous blood was collected from the pregnant females before 15 wk of gestation into vials containing EDTA (S-Monovette EDTA K3E, Sarsted) by trained laboratory technicians. The hemoglobin concentration was measured immediately using the HemoCue Hb 201 System (HemoCue). The blood samples were centrifuged at 2000 *g* for 10 min at room temperature; plasma was separated, aliquoted into cryotubes, and stored at –70°C in the laboratory before shipment to Norway on dry ice for biobanking and further analyses.

### Plasma Se concentration

Analysis was conducted at the Environmental Pollutant Laboratory at the University Hospital of North Norway. Plasma samples (200 μL) were diluted 1:20 with an alkaline reagent by a liquid handler (Freedom Evo 200, Tecan Männedorf) as described previously [36]. Instrumental analysis was performed by inductively coupled plasma mass spectrometry (Nexion 300D, Perkin Elmer) in kinetic energy discrimination mode with helium as cell gas at 4.8 mL flow. Rhodium (Inorganic Ventures) acted as an internal standard. Matrix-matched calibration curves were prepared with ClinCal plasma samples (Recipe Chemicals and Instruments GmbH). Two sets of control samples from Recipe Chemicals, as well as SeroAS (ClinChek plasma and Seronorm serum, both levels 1 and 2), were analyzed together with each batch of 32 study samples and had variation coefficients of 5% and 6%, respectively. The method detection limit for Se with 2.39 μg/L was calculated based on 3 times the SD of Se concentrations detected in the procedure blank samples (*N* = 105), which were prepared and analyzed together with our study samples. All procedures were evaluated for protocol fidelity and handled by trained and qualified laboratory technicians according to the clinical accreditation standard NS-EN IS 15189:2012. There are variations in the reference range and cut-off value of Se throughout the world and the reference range should be established based on the population attributes and dietary patterns of countries [37].We used the Norwegian cut-off of below 71.1 μg/L (0.90 μmol/L) as maternal Se deficiency for ≤18 wk of gestation [16]. For the general population, 80–95 μg/L is suggested to be the reference value of adequate Se plasma concentration [38], which is very close to our set cut-off from a Norwegian pregnant population.

### Early child neurodevelopment

The Bayley-3 was used to assess child neurodevelopment at 3 time points: 6, 12, and 24 mo of age. Bayley-3 is an individually administered tool for children 1–42 mo old consisting of 3 domains: cognitive, language (receptive and expressive communication), and motor (fine and gross motor) [39]. The test takes 45–60 min to administer. In this study, assessments were done in a well-lit room, free from distractions. We ensured that the children were well-fed and not sick before starting the test. We have previously performed cultural adaptations of the Bayley-3 in the same population [[40], [41], [42]] and documented feasibility and excellent inter-rater agreement of the Bayley-3 assessments in the same study setting [42].

Three local psychologists holding a master’s degree in psychology and experienced in conducting Bayley-3 assessments for research performed the assessments under regular interaction and supervision from senior psychologists. All the testers were thoroughly trained and completed standardization exercises with 20 children before the study. For quality assurance during the study period, we randomly selected 8%–10% of the Bayley-3 assessments to be analyzed by the expert assessor. The agreement reached intraclass correlations between 0.84 and 0.99 for the assessments at the different time points, indicating excellent inter-rater agreement. Bayley raw scores were converted to composite scores [expected mean (SD) of 100 (15)] and scaled scores [expected mean (SD) of 10 (3)] based on American norms [43]. For the 6- and 12-mo assessments, we used scores adjusted for preterm birth according to guidelines. For this study, we also calculated a total *z*-score based on the 3 composite scores (that is, cognitive, language, and motor) at each point of assessment as an overall representation of neurodevelopment in children.

### Statistical analysis

All demographic and clinical characteristics of the participating mothers and children are presented in numbers (*N*) and percentages (%), or by means and SD. The missing data were handled using the listwise deletion method. The full sample is used for the maternal Se analysis. With 290 deficient and 509 replete, we had 80% and 90% power to detect differences of 0.21 and 0.24 standardized effect sizes, respectively. In these calculations, we assumed a 2-sided alpha error of 0.05. For the analysis of Se and neurodevelopment, we included dyads with maternal Se data and ≥1 Bayley-3 observation from the 3-time point. We estimated the associations between maternal Se concentrations and Bayley-3 scores at 6, 12, and 24 mo of age using linear mixed models. Se concentration and timepoint for the Bayley-3 assessment (6 mo, 12 mo, and 24 mo) were included as fixed effects, and the individual as a random effect. The primary outcome was predefined as the Bayley-3 total *z*-score, whereas the Bayley-3 composite (cognitive, language, and motor) and subscale (expressive and receptive language, and gross and fine motor) scores were predefined as secondary outcomes. In the mixed models, we also included interaction terms between *1*) sex and Se concentration and *2*) vitamin B_12_ supplementation and Se concentration to assess potential effect measure modification. In these analyses, both the exposure and the outcomes were included as continuous variables. Post hoc, we repeated the analyses using Se concentration categorized as deficient (Se concentration <71.1 μg/L) or not, as well as in tertiles, with the same Bayley-3 outcomes as in the main analyses. All models were adjusted for the WAMI score and maternal age. The adjustment variables were selected based on their potential role as determinants of both maternal Se concentration and neurodevelopment scores (Supplemental Figure 1). Analyses were performed using Stata version 18 (StataCorp).

### Ethics

The original study has ethical approval from the Nepal Health Research Council in Nepal (NHRC; 253/2016, follow-up approval 423/2023) and from the Regional Committee for Medical and Health Research Ethics in Norway (REK vest; reference number 2016/1620). We obtained written informed consent from the mothers after providing thorough information on the study activities.

## Results

At enrollment, the mean age of pregnant mothers was 27.5 y. In total 78.8% had completed grade 10 (secondary school) or above, and 67.1% were working. Among the participants, 67.4% of the families owned land, 24.8% lived in rented houses, and 26.8% of the families had a kitchen and bedroom in the same room (Table 1). Of the children, 74 (9.3%) were born with LBW, 8.8% were born prematurely, and 397 (51.8%) were male (Table 2).

**TABLE 1:** Demographic, socioeconomic, and nutritional features of 800 mothers of Bhaktapur Nepal.

Demographic and socioeconomic features	*N* = 800
Mother’s age, mean **±** SD	27.5 (4.0)
Education	
Mothers who completed secondary school or above	625 (78.8%)
Fathers who completed secondary school or above	600 (75.8%)
Occupation	
Mothers who work	537 (67.1%)
Fathers who work	794 (99.3%)
Caste/ethnicity	
Newar	626 (78.3%)
Brahmin/Chhetri	77 (9.6%)
Indigenous group	75 (9.4%)
Others	22 (2.8%)
Socioeconomic status	
Monthly income^1^	
High (>75 k)	63 (7.9%)
Medium (>25–75 k)	417 (52.1%)
Low (≤25 k)	320 (40.0%)
Family staying in joint family	522 (65.3%)
Family residing in rented house	198 (24.8%)
Kitchen and bedroom in the same room	216 (26.8%)
Family having own land	539 (67.4%)
Receiving remittance from abroad	85 (10.6%)
WAMI^2^**(***n* = 750)	0.66 (0.13)
Nutritional status of mother	
Weight (kg), mean (SD)	55.2 (7.6)
Maternal BMI (kg/m^2^), mean (SD)	23.7 (3.0)
Micronutrient status of mother: selenium and hemoglobin concentrations	
Plasma blood selenium concentration (μg/L) (*n* = 799), mean (SD)	74.8 (10.4)
Low (<71.1 μg/L)	290 (36.3%)
Normal (≥71.1 μg/L)	509 (63.7%)
Median	74.3
(2.5, 97.5)^3^	(56.1, 97.5)
(5, 95)^3^	(58.7, 91.5)
(25, 75)^3^	(66.6, 80.9)
Lowest tertile (*n* = 267)	63.9 (4.7)
Middle tertile (*n* = 266)	74.4 (2.3)
Highest tertile (*n* = 266)	86.1 (7.2)
Plasma Se at gestational week ≤12 wk, mean (SD) (*n* = 420)	76.1 (10.2)
Plasma Se at gestational week after 12 wk, mean (SD) (*n* = 334)	73.2 (10.1)
Hemoglobin concentration (g/dL) (*n* = 799), mean (SD)	12.8 (1.2)

All numbers are numbers (percentage) if not otherwise stated.

Abbreviation: WAMI, Water and sanitation, Asset ownership, Maternal education, and household Income.

1 Nepalese rupees.

2 Water and sanitation, household assets, maternal education, and household income, range of 0–1.

3 Percentiles.

**TABLE 2:** Characteristics of 767 newborns in Bhaktapur Nepal.

Infant’s characteristics	*N* (%)
Male	397 (51.8)
Low-birth weight (<2500 g)	74 (9.3)
Preterm birth (<37 wk of gestation)	70 (8.8)

The mean plasma Se concentration of pregnant females was 74.8 μg/L (±10.4) and 36.3% had Se concentration below the suggested cut-off of 71.1 μg/L indicating deficiency (Table 1). The 2.5th and the 97.5th percentiles Se concentrations were 56.1 μg/L and 97.5 μg/L, respectively.

Table 3 describes the estimated associations between maternal Se concentration and Bayley scores using linear mixed models. There were no linear associations between maternal Se concentration and the Bayley total *z*-score [*β* = 0.002 (95% confidence interval [CI]): crude: –0.003, 0.007]; coeff. 0.002 (95% CI: adjusted: –0.007, 0.011). Furthermore, there were no linear associations between maternal Se concentration and Bayley-3 composite or the scaled scores (Table 3). Neither sex of the child nor maternal vitamin B_12_ supplementation significantly modified the associations between Se concentration and neurodevelopmental scores [sex: *β* = 0.002 (95% CI: adjusted –0.007, 0.011); vitamin B_12_: *β* = 0.001 (95% CI: adjusted –0.007, 0.008)]. Analyzing Se concentration in both dichotomized (Se deficient compared with normal) (Table 3) and tertiles in the mixed model confirmed the main findings of no association between Se concentration and Bayley-3 scores (Table 4).

**TABLE 3:** Associations between maternal plasma selenium concentration and Bayley scores of children of Bhaktapur Nepal at 6, 12, and 24 mo using linear mixed model over time.

Bayley scores	Se exposure	Crude			Adjusted		
		Coeff.	95% CI	*P* value	Coeff.	95% CI	*P* value
*z*-total	Continuous	0.002	–0.003, 0.007	0.496	0.002	–0.007,0.011	0.628
	Deficient^1^	Ref.			Ref.		
	Normal	0.078	–0.032, 0.188	0.165	–0.119	–0.285, 0.047	0.061
Cognitive composite	Continuous	0.026	–0.020, 0.072	0.273	0.050	–0.040, 0.127	0.270
	Deficient	Ref.			Ref.		
	Normal	–0.089	–0.192, 0.013	0.087	–0.103	–0.259, 0.053	0.197
Language composite	Continuous	0.038	–0.015, 0.091	0.164	0.010	–0.080, 0.101	0.820
	Deficient	Ref.			Ref.		
	Normal	–0.107	–0.279,0.065	0.222	–0.098	–0.361, 0.165	0.465
Receptive scaled		0.004	–0.006, 0.015	0.396	0.001	–0.017, 0.018	0.952
Expressive scaled		0.008	–0.002, 0.019	0.123	0.004	–0.014, 0.023	0.644
Motor composite	Continuous	–0.033	–0.095, 0.028	0.288	–0.030	–0.134, 0.079	0.613
	Deficient	Ref.			Ref.		
	Normal	–0.007	–0.115, 0.101	0.898	–0.120	–0.283, 0.043	0.149
Fine motor scaled		–0.005	–0.017, 0.007	0.415	0.000	–0.020, 0.021	0.980
Gross motor scaled		–0.007	–0.019, 0.004	0.228	–0.008	–0.029, 0.011	0.400

Abbreviations: CI, confidence interval; WAMI, Water and sanitation, Asset ownership, Maternal education, and household Income.

1 Selenium concentration <71.1 μg/L. Adjusted for maternal age, sex of the child, WAMI, and vitamin B_12_ treatment.

**TABLE 4:** Associations between maternal plasma selenium concentration (tertiles) and Bayley scores of children of Bhaktapur Nepal at 6, 12, and 24 mo using linear mixed model over time.

Bayley scores	Se concentration	Crude			Adjusted		
		Coeff.	95% CI	*P* value	Coeff.	95% CI	*P* value
*z*-total							
	Lowest tertile	–0.072	–0.202, 0.057	0.272	–0.104	–0.273, 0.066	0.231
	Middle tertile^1^	Ref.			Ref.		
	Highest tertile	–0.009	–0.138, 0.121	0.895	0.014	–0.165, 0.193	0.877
Cognitive composite							
	Lowest tertile	–0.105	–0.226, 0.015	0.086	–0.129	–0.382, 0.123	0.316
	Middle tertile^1^	Ref.			Ref.		
	Highest tertile	–0.014	–0.135, 0.107	0.818	–0.005	–0.163, 0.173	0.952
Language composite							
	Lowest tertile	–0.069	–0.272, 0.133	0.499	–0.042	–0.311, 0.226	0.759
	Middle tertile^1^	Ref.			Ref.		
	Highest tertile	0.024	–0.179, 0.226	0.820	–0.017	–0.301, 0.266	0.906
Motor composite							
	Lowest tertile	–0.022	–0.148, 0.105	0.739	–0.102	–0.269, 0.064	0.229
	Middle tertile^1^	Ref.			Ref.		
	Highest tertile	–0.051	–0.178, 0.075	0.428	–0.021	–0.155, 0.197	0.813

Adjusted for maternal age, sex of the child, WAMI, and vitamin B_12_ treatment.

Abbreviations: CI, confidence interval; WAMI, Water and sanitation, Asset ownership, Maternal education, and household Income.

1 Reference group.

## Discussion

Although Se is considered a vital micronutrient for brain development and 36% of the mothers had low Se concentration (<71.1 μg/L), we did not find any association between maternal Se plasma concentration and neurodevelopment of children measured aged at 6, 12, and 24 mo in a population-based study in Bhaktapur, Nepal.

About one-third of the pregnant females had Se deficiency during the early weeks of pregnancy. This confirms previous findings of Se deficiency in the soil of neighboring parts of Tibet and soil leaching conditions in Nepal, resulting in the depletion of essential nutrients, including Se for plants [44]. This may possibly cause Se deficiency in the population. Moreover, the plasma Se concentrations in the current study are similar to those found in a population of Norwegian pregnant females [16]. However, in a study from Bangladesh, ∼60% of the participants were deficient when applying a 60 μg/L cut-off [45] which is considerably above the prevalence of deficiency observed in our Nepalese population. Of note, the current cohort is from a healthy population with specific exclusion criteria for the study.

Our findings of no significant association between Se concentrations in pregnant females and infant neurodevelopment are in discrepancy with other studies [[16], [17], [18], [19],^21^,^22^]. A possible explanation is that there were few cases of both extreme Se deficiency and high Se concentration in our healthy cohort. Notably, the previous studies with significant positive findings had different population characteristics especially with either higher maternal Se concentrations [17,20] or lower maternal Se concentration compared with the current study [18,21,46], except from the Norwegian study [16]. The range of Se plasma concentration in our study was 47–127 μg/L. This distribution shows no excessive Se concentrations based on reference values of 90–120 μg/L [47] or 70–155 μg/L [48] which could be considered a safe range for human health and neurotoxicity during the prenatal phase [24]. Moreover, Se plasma concentration in the range of 80–95 μg/L is considered optimal for enzyme functions [38], which is close to our cut-off, justifying that our sample is in the safe range.

We could not find support for sex as a moderator for the association. This is in contrast to the results from Bangladesh and China where studies found differential associations according to the sex of the child [20,22]. We did not find evidence of a modifying effect of vitamin B_12_ supplementation in the association between plasma Se and Bayley-3 total score. It is important to note that our findings of no association, which contrast with most other studies, can be explained by several factors such as confounding bias and effect modification. For instance, Se concentration may be linked to socioeconomic status in some populations but not in others. Socioeconomic status is typically associated with developmental scores and could act as a confounder. Additionally, in certain low socioeconomic populations, poor Se status might have no effect due to other, more critical determinants of neurodevelopment.

The main strength of the study is the relatively large sample size and repeated high-quality assessments with the Bayley-3 at 3 time points using standardized methods and trained professionals showing high inter-rater agreement both in the standardization exercises and in the conduct of the study. There were good variabilities of the Bayley total scores across the 3 time points, and because the scores were normally distributed it would be possible to pick up differences between participants (Supplemental Figure 2). Laboratory procedures for plasma sampling and Se analysis were carried out by trained and qualified personnel. Samples were stored according to biobank standards. The Se analysis was fully based on appropriate recommended procedures that have established rigor in our assessment. Despite including Se as flexible, linear, and categorical variables in the models, we found no significant association between maternal concentrations of Se and the child’s Bayley-3 total or subscale scores. Although Bayley-3 is the reference standard for neurodevelopmental assessment, more specific cognitive functions are better assessed at later time points with neuropsychological measures. Thus, future studies should explore associations with specific cognitive outcomes.

The study also has several limitations. Plasma Se concentration may not be an optimal biomarker for Se status [49] which could lead to random misclassification. Such misclassification could both underestimate the association between Se and Bayley-3 scores and overestimate deficiency [50]. Still, previous studies have found an association between plasma or serum Se and clinical and functional outcomes [[16], [17], [18], [19], [20], [21], [22],^51^]. Of note, the current cohort is from a healthy population with specific exclusion criteria, limiting the generalizability of results. We could have used raw scores for our data presentation, but because raw scores would not be comparable across age groups and time points and standardized scores communicate the findings more clearly, we have used standardized scores with an expected mean (SD) of 10 (3) or 100 (15) for the scaled or composite scores. We have previously verified the feasibility of Bayley-3 in a Nepalese setting with cultural adaptations [42]; we do not, however, have Nepalese representative sample norms and hence used American sample norms for the conversion of the Bayley raw scores into scaled and composite scores. Although there were no associations between maternal Se concentration and Bayley-3 scores which are assessed based on specific skills and tasks, this finding does not rule out the possibility of differences in underlying neural processes that could be assessed through brain scans such as fMRI. A multimodal approach to understand brain function is recommended [52,53]. Furthermore, neuropsychological processing or domain-specific measures later in childhood can give insight into more specific cognitive functions.

In conclusion, about one-third of pregnant females in a cohort of healthy Nepalese females had biochemical signs of Se deficiency during the first and early phase of the second trimester. In contrast to most/many other studies, we did not find any significant association between maternal Se concentration in early pregnancy and early child neurodevelopment. It is important to identify the consequences of population-based Se deficiency, appropriate biomarkers, and optimal cutoffs in various populations.

## Author contributions

The authors’ responsibilities were as follows – SR, RKC, TAS, IK, MU, MH: designed research; SR, IK, RKC, TAS, MH: analyzed data, primary responsibility for final content; SH, MA: analyzed blood samples; and all authors: conducted research, wrote the paper, and read and approved the final manuscript.

## Data availability

Data are available on request. The request must be first approved by the Nepal Health Research Council, Nepal, and should be sent to the authors by sending an email to the Department of Pediatrics, Institute of Medicine, Tribhuvan University, Kathmandu, Nepal (chrp2015@gmail.com).

## Funding

This study is supported by Research Council of Norway project grant number 336566.

## Conflict of interest

The authors report no conflicts of interest.

## Appendix A Supplementary data

Multimedia component 1 Supplementary Figure 1. Directed Acyclic Graph of the association between maternal selenium and Bayley-3 scores controlling for socioeconomic status (WAMI) and maternal age Supplementary Figure 2. Bayley total z-score calculated based on 3 composite scores. Multimedia component 1
