# Supplementary material for: The Association Between Prenatal Maternal Selenium Concentration and Neurodevelopment in Early Childhood, Results from a Mother–Child Cohort Study
Source: J Nutr. 2025 Apr 11;155(6):1962–9. doi: 10.1016/j.tjnut.2025.04.005 (PMC12264534; doi:10.1016/j.tjnut.2025.04.005)

**Supplementary Materials**

**The association between prenatal maternal Selenium concentration and neurodevelopment in early childhood, results from a mother-child cohort study**

(Ranjitkar et al.)

**Supplementary Table 1:** **Calculation of the WAMI index**

| **Component** | **Description** | **Score range** | |
| --- | --- | --- | --- |
| **W**ater and Sanitation | Using the World Health Organization definitions of access to improved water and improved sanitation, households with access to safe water or safe sanitation were assigned a score of 4 for each. For this context, bottled water/jar, water from water tanks and tap water supply were considered as improved. Households without access to improved water or improved sanitation (well, hand pump and other sources) were assigned a score of 0 for each. | 0-8 |  |
| **A**ssets | For each asset, households were assigned a 1 if they had the assets and a 0 if they did not have the asset. The following assets were used: used ownership land, bedroom/kitchen separate, ownership of motorbike, TV, Fridge, Personal computer, microwave oven and car. | 0-8 |  |
| **M**aternal education | Here the maximum year of schooling is 18. Maternal education was assigned first dividing the total years of schooling by 2, then multiply by 8/9. | 0-8 |  |
| **I**ncome | Total household income is presented in octiles. | 0-8 |  |
|  |  |  |  |
| Total WAMI | Scores in water and sanitation, assets, maternal education, and income were summed then divided by 32. | 0-1 |  |

**Supplementary Figure 1**


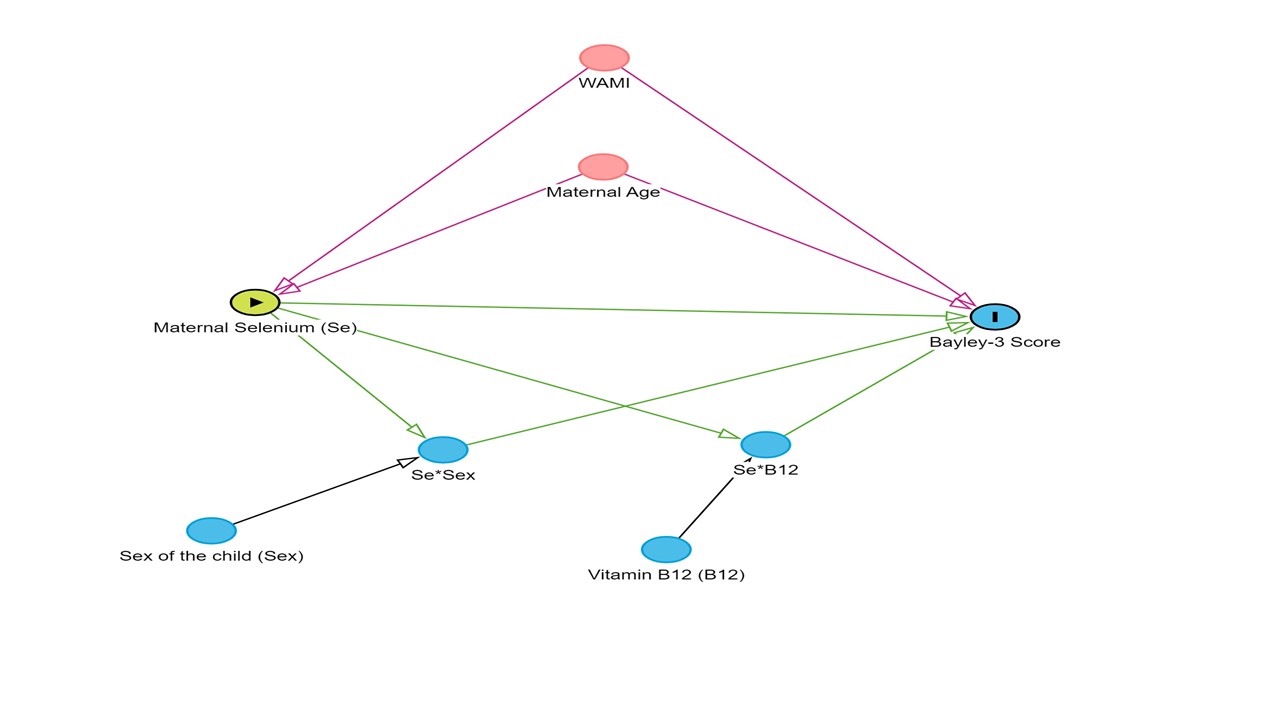


**Supplementary Figure 2**


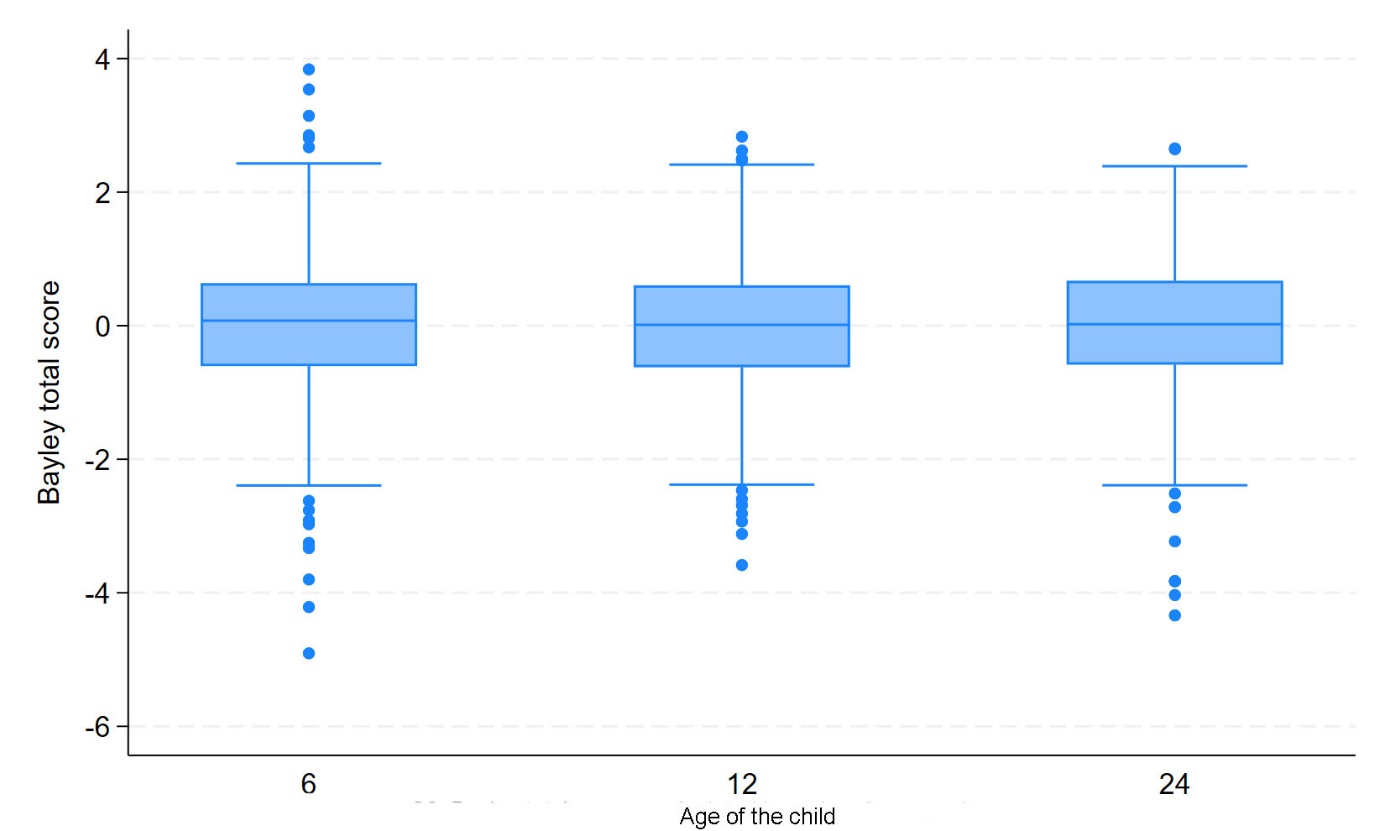

Supplement: Multimedia component 1 — Supplementary Figure 1. Directed Acyclic Graph of the association between maternal selenium and Bayley-3 scores controlling for socioeconomic status (WAMI) and maternal age Supplementary Figure 2. Bayley total z-score calculated based on 3 composite scores. [file mmc1.docx]
